# Major Depressive Disorder and Alterations in Insular Cortical Activity: A Review of Current Functional Magnetic Imaging Research

**DOI:** 10.3389/fnhum.2012.00323

**Published:** 2012-12-03

**Authors:** Diane Sliz, Shawn Hayley

**Affiliations:** ^1^Department of Neuroscience, Carleton UniversityOttawa, ON, Canada

**Keywords:** insula, fMRI, depression, interoception, awareness, stress, DMN, neuroplasticity

## Abstract

Major depressive disorder (MDD) is characterized by a dysregulated fronto-limbic network. The hyperactivation of limbic regions leads to increased attention and processing of emotional information, with a bias toward negative stimuli. Pathological ruminative behavior is a common symptom of depressive disorder whereby the individual is unable to disengage from internal mental processing of emotionally salient events. In fact, lower deactivations of the neural baseline resting state may account for the increased internal self-focus. The insular cortex, with its extensive connections to fronto-limbic and association areas has recently also been implicated to be a part of this network. Given its wide-reaching connectivity, it has been putatively implicated as an integration center of autonomic, visceromotor, emotional, and interoceptive information. The following paper will review recent imaging findings of altered insular function and connectivity in depressive pathology.

## Major Depressive Disorder

According to the World Health Organization, major depressive disorder (MDD) will be the second-leading cause of disability in the world by the year 2020, following heart disease (Möller and Henkel, 2005). The Canadian Mental Health Association predicts that one in 10 individuals will suffer from depression during their lifetime, whereby approximately 3.3 million people will go through at least one depressive episode (Winn and Laufer, 2001). The National Co-morbidity Survey reported that 58% of patients with major depression also fulfill criteria for an anxiety disorder (Kessler et al., 2005). Depression and anxiety share many overlapping symptoms including fatigue, impaired concentration, irritability, sleep disturbances, and somatization in addition to subjective experiences of nervousness, worry, and restlessness (Ressler and Nemeroff, 2001).

A myriad of scientific studies have examined the neural circuitry involved in emotional regulation, especially as it applies to mood disorders. It is believed that a dysregulation in brain regions associated with affect are central in the etiology of MDD (Kring and Bachorowski, 1999). There is also evidence for abnormalities of the norepinephrine (NE) and serotonin neurotransmitter systems in depression and anxiety disorders. Dysfunction of these neurotransmitters is likely due to their role in modulating and being modulated by other neurobiological systems that together mediate the symptoms of the affective illness.

Brain imaging studies support the view that fronto-limbic networks are dysregulated in major depression. Indeed, reductions in gray matter density (Frodl et al., 2008) and volume (Vasic et al., 2008; Lai et al., 2010) as well as altered activity have been noted in hippocampal, amygdala, cingulate, and prefronto-cortical regions (Mayberg, 1997; Lee et al., 2008). The morphological changes in these brain networks likely engender impairments in cognitive and affective processing given the reduced neuroplasticity and hence disrupted connectivity amongst these brain regions (Fossati et al., 2004). Substantial research has shown that behavioral deficits can be improved and brain circuits can be normalized (Ressler and Mayberg, 2007) using pharmacological (Delaveau et al., 2011) as well as non-pharmacological approaches such as deep brain stimulation (Mayberg et al., 2005), repetitive transcranial magnetic stimulation (TMS; Li et al., 2004), and cognitive behavioral therapy (Goldapple et al., 2004).

Recent imaging research suggests an altered basal neural resting state in MDD patients, characterized by increased neural activity in certain brain regions encompassing the default mode network (DMN; Lemogne et al., 2012; Zhu et al., 2012). The inability to down-regulate activity within the DMN may explain the varied symptoms in depression, namely, increased self-focus (Sheline et al., 2010), impaired attentional control (Marchetti et al., 2012), and maladaptive rumination (Hamilton et al., 2011). The right fronto-insular cortex has been linked to greater depressive rumination (Hamilton et al., 2011) in depressed patients, implicating this region in the abnormal increased self-focus commonly seen in populations with mood disorders (Grimm et al., 2011).

The ensuing sections will review behavioral markers and neurochemical alterations in MDD. As well, the neural correlates of MDD in the context of disrupted fronto-limbic connectivity will be discussed. Recently, the brain’s resting state (i.e., DMN) has garnered increasing attention as a dysregulated network in MDD. As the focus of this paper, the insular cortex, a region reported to be a part of the DMN and an important cortical structure implicated in emotional processing and regulation (among other functions) will be further examined in the context of MDD. Finally, recent imaging findings will be presented contrasting the role of the insula in depression across different information processing paradigms (cognitive, social, emotional, and memory systems). As well, the insula’s role in the resting state and following therapeutic treatment will be examined.

### Behavioral, neurochemical, and neural markers

Emotional self-regulation is hindered in individuals suffering from MDD, causing a disruption in their attentional focus and creating memory biases toward negative life events. This results in an altered mood state due to negative sensory representations, which consequently modify cognitive processes (Siegle et al., 2002). Depression is further characterized by an anhedonic state (inability to experience pleasure), cognitive inhibition, and withdrawal tendencies (Stein, 2008; Gohier et al., 2009).

Several meta-analyses have highlighted the role of the insula in depression with differing results. Hamilton et al. (2011) found that depressive populations respond to negative information to a greater degree, as it is more salient to them. The insula has been posited to play a role in this salience network (Hamilton et al., 2011), whereby incoming sensory information is being misinterpreted (Palaniyappan and Liddle, 2012) leading to abnormal responses (e.g., judging a neutral facial expression as negative). Contrary to these findings, Fitzgerald et al. (2008) found hypoactivity in insular, cingulate, and frontal regions in resting state paradigms, treatment studies as well as negative emotion induction studies. These differences may be explained by differing population samples. While both groups examined individuals diagnosed with depression, it is unclear whether individuals had comorbid disorders, were medicated, suffered from a first episode or were chronic patients, paradigms used, etc. All of these factors play a role in the interpretability of the results presented above.

Emotional regulation studies seem to provide a more consistent view of the insula’s role in depressive disorder. A disruption in top-down processing is thought to mediate pervasive feelings of sadness and negative affect in depressed populations, whereby dorsolateral prefrontal and inferior parietal cortex activity is decreased and subgenual cingulate and anterior insula activity is increased (Mayberg et al., 1999). The altered connectivity among these regions likely leads to inefficient regulation and response to negative mood. Perlman et al. (2012) demonstrated greater emotional reactivity in a group of un-medicated adolescents when prompted to maintain their reactions to negative images. This activity increased right insula activation and showed decreased connectivity between the amygdala and insular/prefrontal regions, indicating poor top-down emotional regulation (Perlman et al., 2012). Indeed, greater activity has been detected in the insula (among other limbic regions) when trying to suppress sadness (Beauregard et al., 2006) and reappraise a negative stimulus (Johnstone et al., 2007). In fact, greater use of expressive suppression (an emotional regulation strategy that inhibits the outward expression of an internally generated emotion) has been linked to greater insula volume in healthy adults, suggesting greater control over emotionally relevant information (Giuliani et al., 2011).

Several studies have linked insular function to depressive symptoms and behaviors. A principal component analysis of the Beck Depression Inventory revealed that psychomotor anhedonia symptom severity, characterized by decreased satisfaction and loss of interest in others, was negatively correlated with metabolism in the right insula (Dunn et al., 2002). Dysfunction in the insula has been noted for facial expressions (reviewed by Stuhrmann et al., 2011), and has also shown to be positively correlated with the Hamilton Depression Rating Scale (HDRS) in response to negatively valenced images (Lee et al., 2007). However, treatment resistant female depressed patients show greater insula activity in response to positively valenced pictures (Mitterschiffthaler et al., 2003). Interestingly, treatment naïve MDD patients were found to exhibit a negative correlation between insula cerebral blood flow and agitation, as well as weight loss (items on the HDRS). Recently, a review by Mutschler et al. (2012) revealed that emotional processing in depressed people is shifted to the dorsal insula (a region involved in pain processing in healthy adults), thus providing a possible neural correlate for emotional allodynia (emotional pain) in depressed populations.

Given that hereditary factors are often implicated in mood disorders, it should not be surprising that offspring of parents diagnosed with depression show altered neural functioning in mood processing and regulating brain areas. Using an incentive delay task whereby prizes are awarded upon hitting a target, Gotlib et al. (2010) showed that young daughters of mothers with a history of MDD (termed high-risk) had greater activation in the right insula in response to anticipation of reward compared to young daughters with healthy mothers. The different activation patterns in the insula in high-risk daughters show evidence of a different perception or interpretation in response to reward and possibly a higher proclivity of developing a mood disorder. McCabe et al. (2012) found strikingly similar results when examining healthy adolescents with one depressed parent. Increased activation in the insula was detected following the administration of an aversive taste as well as an unpleasant sight (rotten strawberry). This population was, however, unable to activate similar brain regions (posterior insula) in response to a pleasant tasting chocolate stimulus, indicating an inability to process reward (McCabe et al., 2012).

It is plausible that a negative self-image (Northoff, 2007) and maladaptive thought patterns may generate a “negative filter” where all information from the environment is processed and integrated in a dysfunctional fashion. In fact, a “hate” circuit has been proposed, involving the insula, which would involve its reduced connectivity with the putamen and superior frontal gyrus in both first episode and treatment resistant depressed patients (Tao et al., 2011). Given the insula’s role in self-awareness and integration of visceral and sensory information (further discussed in ensuing sections), this decoupling may reflect disrupted interoception and an inability to exert cognitive control over emotionally relevant information.

In major depression and in general anxiety states, the NE and serotonin (5-HT) systems are clearly imbalanced, with substantial data indicating that NE responses are increased to stressor exposure and fearful stimuli, whereas the 5-HT system is hypo-responsive. These neurotransmitter alterations contribute to the over-activation of the amygdala, hippocampal, and cortical pathways activating stress and fear responsiveness (Ressler and Nemeroff, 2001). Typically in stressful circumstances, abnormally activated central amygdala, paraventricular nucleus of the hypothalamus and bed nucleus of the stria terminalis lead to increased release of corticotrophin releasing factor (CRF) and adrenal steroids together with increased activity of autonomic, visceral, and neural pathways associated with the stress/fear response. Moreover, CRF has repeatedly been reported to be elevated in the cerebrospinal fluid of patients with depression (Plotsky et al., 1998). Similarly, up to 75% of patients with major depression have an overactive hypothalamic pituitary adrenal (HPA) axis as characterized by hypercortisolemia. In fact, it has even been hypothesized that stress, depression, and perhaps certain anxiety disorders are associated with degeneration of target neurons, perhaps mediated by cortisol hypersecretion and chronic levels of elevated adrenal glucocorticoids, contributing to hippocampal and cortical atrophy (Duman et al., 1999; McEwen, 1999). Decreased neuronal density and atrophy of the hippocampus presumably contributes to decreased hippocampal activity, possibly diminishing its ability to inhibit amygdala stressor and fear responsiveness.

Atrophy of the prefrontal cortex likely decreases the ability of cortical modulation and inhibition of amygdala aversion pathways. It has been proposed that the prefrontal cortex is involved via connections with the amygdala in regulating affect, providing cognitive control over stress and fear responsiveness along with anger, anxiety, and frustration tolerance (Lang et al., 1998). Decreased 5-HT release may also contribute to this neuronal degeneration, dendritic atrophy, and lack of regeneration by diminished neurotrophic factor release. Indeed, the combined shifts of the NE and 5-HT neurotransmitter systems in depression lead limbic pathways to prevail over prefrontal cortical pathways in the control of affect. More specifically, NE tends to increase memories of aversive events, and to be underactive in higher cortical areas, such as the prefrontal cortex, which is critical in mediating the extinction of fearful memories. Thus, the same intensity of stressful stimuli that might only lead to minimally increased arousal in the normal euthymic state, might lead to significant arousal, vigilance, and activation of fearfulness in depressed patients (Ressler and Nemeroff, 2001). Together, these processes likely serve to maintain a state of neurochemical imbalance by decreasing the ability of cortical and hippocampal areas to inhibit and modulate the stress/fear pathways of the amygdala and interconnected circuitry.

Serotonergic synapses are most densely concentrated in limbic regions including the amygdala and the BNST, as well as the ventral striatum and hypothalamus. Serotonin from the raphe nuclei mediates tolerance to aversive experience in the amygdala, potentially decreasing the likelihood of a fight or flight response (Ressler and Nemeroff, 2001). There is also increasing evidence that 5-HT activates neurotrophic factor activity in various brain regions, that seems to be decreased in response to stressors and in depression (Duman et al., 1999). In fact, reductions in serotonin receptor binding in the insula among other regions have been found in individuals suffering from social anxiety disorder (Lanzenberger et al., 2012), which may explain anxious and depressive-like symptoms in this population. Conversely, Cannon et al. (2007) showed increased binding potential of the serotonin transporter in the insula, thalamus, and striatum of both bipolar and depressive populations compared to healthy controls. These discrepant findings may be attributed to differences in clinical samples.

Other neurotransmitters have also been implicated in insula activity. For instance, reduced binding of a metabotropic glutamate receptor (mGluR5) was observed in the insula, cingulate, and hippocampal brain regions in depressed patients compared to healthy controls (Deschwanden et al., 2011). In fibromyalgia patients (a condition characterized by fatigue and widespread pain), GABA (major inhibitory neurotransmitter) levels were significantly decreased in the right anterior insula compared to healthy controls (Foerster et al., 2012). This may provide insight into a disrupted inhibitory neurotransmission in the right portion of the anterior insula, which is thought to coordinate information from the parasympathetic system (Craig, 2009).

Neuroanatomically, depressive disorders are associated with changes in the emotional circuitry, encompassing the limbic structures as well as the prefrontal and parietal cortices. The amygdala, in association with other fronto-limbic structures, plays a pivotal role in the processing of emotional stimuli and arbitrates emotional influences in other brain regions related to attention, memory, and decision-making (Hariri et al., 2000; Wang et al., 2006). The amygdala also has a visceral function and its response is dependent on the relevance of the stimulus to the individual’s current behavioral state (Wang et al., 2006). Therefore, a depressive state would yield greater activation in the amygdala and associated regions, in response to negative affect. The emotional circuitry in the limbic system outputs from the hippocampal formation to the mamillary body, passing via the anterior thalamus nuclei and extending to the cingulate gyrus, which is the receptive region for the experience of emotion (reviewed by Morgane et al., 2005). This pathway, in turn, communicates with the hypothalamus, which provides output in the form of autonomic functions and neuroendocrine factors provide a physiological expression of emotional states (Beauregard et al., 2001; Morgane et al., 2005).

Ochsner et al. (2002) illustrated that the neural basis for the cognitive control of emotion extends to the prefrontal cortex, the medial orbitofrontal cortex as well as the amygdala. The latter two structures are involved in emotional processing, with the prefrontal cortex playing a role in awareness, executive functions, behavioral inhibition, attention, and working memory (Ochsner et al., 2002; Morgane et al., 2005), all of which are found to be impaired in MDD (Yorbik et al., 2004; Gohier et al., 2009). By examining how individuals reappraise highly negative scenes, Ochsner et al. (2002) was able to illustrate that the cognitive transformation of an emotional experience (i.e., negative photos) decreases negative affect in healthy participants. Consequently, reappraisal may modulate the emotional processes employed in the amygdala and medial orbitofrontal cortex, which are involved in the evaluation of the affective salience and contextual relevance of the stimulus (Ochsner et al., 2002). These findings suggest that the intricate connections between the amygdala and prefrontal cortex, allow the frontal structures to act as top-down modulators of the emotional response in the amygdala (Beauregard et al., 2001; Bermpohl et al., 2006). In fact, cortical connections from the amygdala and other limbic structures (e.g., parahippocampal formation) are directed to the orbito- and medial prefrontal cortex, which interacts with the thalamus and basal ganglia. This pathway forms a circuit involved in the stimulus-reward association, reward-guided behavior, and mood determination (Price, 1999).

In addition to the functional alterations in the emotional processing and regulation centers of the brain, structural abnormalities have also been noted in hippocampal and prefrontal regions (see reviews by Fossati et al., 2004; Drevets et al., 2008; and meta-analysis by Koolschijn et al., 2009). These morphological changes may account for the disrupted connectivity between limbic structures and higher-order processing which modulate these autonomic and immediate affective states. Indeed, coupled with sensitization to stressors (via a hyperactive HPA axis), glucocorticoids, which typically provide a negative feedback loop, are pathologically overexpressed in depression (reviewed by Sapolsky, 2000; Tata and Anderson, 2010) and have been linked to neuronal death and hippocampal atrophy (reviewed by Lupien et al., 2007; Fales et al., 2008).

## Default Mode Network

### Defining the DMN

The DMN has been defined as the baseline neural activity when one is disengaged from externally cued cognitive demands (Gusnard and Raichle, 2001; Greicius et al., 2003). Using functional connectivity magnetic resonance imaging (fMRI), the DMN has been found to constitute the following primary brain regions: posterior/precuneus and ventral anterior cingulate cortices, dorsolateral and medial prefrontal cortex, inferior parietal cortex, superior temporal gyrus, orbitofrontal cortex, and parahippocampal gyrus (Raichle et al., 2001; Greicius et al., 2003; Raichle and Gusnard, 2005). The blood oxygen level dependent (BOLD) signals from these anatomically separated brain regions have a high degree of temporal correlation, illustrating an organized neural network which shows increased activation during the resting state (Skudlarski et al., 2008; Greicius et al., 2009). In addition to these regions, the insula has also been implicated in stimulus-independent thought and, as will be discussed shortly, is believed to be a primary node in the DMN (Cauda et al., 2011).

The activities of the DMN have been linked to intrinsic processing (i.e., monitoring of one’s internal and external state Gusnard and Raichle, 2001) and self-referential processing (Schneider et al., 2008). Moreover, it has been described as a system that allows conscious retrieval of past events in order to perform higher-order cognitive functions (i.e., problem solving, planning, decision-making Greicius and Menon, 2004). As well, the resting state has also been attributed to attentional systems, interoception, and emotional processing (Wiens, 2005; Schneider et al., 2008). Importantly, as briefly mentioned earlier, the insula is a key regulatory node in the DMN system and it is likely that altered insular functioning could contribute to disturbances in the internal regulation of autonomic and other aspects of emotional stimuli.

### Insular cortex

Early studies using cortical stimulation techniques in humans and primates have elucidated the role of the insula with visceral phenomena, including abdominal sensations, gastric motility, respiratory, and cardiovascular functions (Penfield and Faulk, 1955; Showers and Lauer, 1961). As well, stimulation of the rostrodorsal and caudoventral portions of the insular cortex has been linked to gross body movement (Showers and Lauer, 1961). Given its neuroanatomical connections with the prefrontal, temporal, parietal, and limbic regions, the insula is also considered an integration center between the external environment and the internal processing and regulation regions of the brain (Mesulam and Mufson, 1982; Mufson and Mesulam, 1982). Recent research has also implicated the insula in auditory processing (Bamiou et al., 2003) as well as taste perception and related gustatory functions (Small, 2010). Insular activation (right anterior portion) has also been found to be positively correlated with disgust sensitivity scores in healthy adults when viewing unpleasant stimuli such as body wounds and cockroaches (Mataix-Cols et al., 2008). These findings extend to depressed populations which seem to be more susceptible to such stimuli. Surguladze et al. (2010), in fact, found increased activation in the left and bilateral insula when viewing facial expressions of disgust presented at 50 and 100% intensity, respectively.

Recently, the insula has garnered increasing attention as being involved in conscious awareness, whereby it has been inferred to hold a representation of one’s current subjective feeling of their body’s physiological state (Craig, 2009). This notion implies a neural substrate for the integration of interoceptive information from the body and the emotional valence that we attribute to these subjective experiences. Brain laterality seems to be important in this process as well, with the right anterior insular cortex responding to arousing stimuli and autonomic sensations, such as pain, while the left anterior insula is activated by sensory salient and emotional feelings (Critchley et al., 2000; Leone et al., 2006; Ortigue et al., 2007; Wiech et al., 2010).

Emerging evidence from neuroimaging studies further lend support to the function of the insular cortex as a hub of meta-awareness, general arousal, and interoceptive processing (Bluhm et al., 2011). While the insula was not detected as one of the “core” regions of the DMN in earlier pioneering work on the brain’s resting state (Gusnard and Raichle, 2001), recent studies reveal that the insular cortex does deactivate but in response to demanding tasks requiring focused attention (Tomasi et al., 2007). Harrison et al. (2005) showed this using the Stroop color-word paradigm. In congruent conditions, words representing one of four colors (red, green, yellow, and blue) were printed in that color (e.g., the word “red” was printed in a red font color). Whereas in incongruent conditions, the meaning of the word was printed with a different color (e.g., the word “red” was printed in a green font color). Interestingly, when comparing resting blocks to an incongruent Stroop condition (vs. a congruent Stroop task), the posterior insula deactivated to a greater degree in the incongruent Stroop condition (Harrison et al., 2005). Having to actively process both the word color and the color with which the word is written causes much greater interference and thus, increased attention and cognitive energy demands need to be allocated toward this task. This suggests that as the cognitive demands on the system increase, the brain regions involved in different elements of subjective awareness and consciousness will deactivate to a greater extent (Harrison et al., 2005; Tomasi et al., 2007; Mayer et al., 2010).

The insula has been further reported to be part of a “salience network” along with the anterior and mid-cingulate cortex (Sridharan et al., 2008; Taylor et al., 2009). The strong coupling between these regions has been posited to play a role in monitoring the emotional salience of stimuli (both interoceptive and exteroceptive) with the aim of integrating this information to form a subjective representation of one’s bodily state (Taylor et al., 2009). As such, the insula acts as the monitor of emotional stimuli of this salience network and the cingulate is engaged and called into action when one responds to the salient information. Sridharan et al. (2008) have further extended the salience network to include the dorsolateral prefrontal cortex (dlPFC) which plays an important role in switching one’s attention between the DMN and the brain’s executive network.

### Dysregulated connectivity of the DMN in major depressive disorder

Neuroimaging studies have provided much evidence for a pathological activation of the brain’s limbic circuits (e.g., amygdala, anterior insular cortex) in depressed individuals in response to emotional events (Drevets, 1999, 2001). Also involved are executive brain regions (medial and dlPFC, orbitofrontal cortex), which modulate these emotionally salient behaviors as well as the autonomic and endocrine responses to stressors (Drevets, 1999, 2001). These neural networks have been shown to be impaired in MDD patients (Mayberg, 1997), wherein the lack of cortical inhibition from limbic input leads to a greater propensity to ruminate and be increasingly aware of internally focused cognitions (Cooney et al., 2010).

Default mode networks have further been investigated as impaired neural targets in MDD pathology. As mentioned previously, the brain’s “resting circuitry” involving cortical midline structures (e.g., vmPFC, pACC, PCC) serves as a neural baseline of task-independent activity, showing greater activation in response to non-demanding self-related processing [e.g., reflection and unconstrained mental activity (mind wandering); Kjaer et al., 2002; Holt et al., 2011; Yan et al., 2009]. In line with the hypothesis that cortical midline structures are implicated in increased association and responsivity to negative emotions (Northoff, 2007), several imaging studies using emotional and cognitive-based paradigms have provided support for the notion of atypical self-focus and introspection in these brain regions.

Using positive and negatively valenced pictures, Grimm et al. (2009a,b) demonstrated that MDD patients show an abnormality in attributing emotions to themselves, particularly when these are negative in nature. MDD patients rated negative pictures as more self-related which was paralled with reduced signal changes in cortical midline structures (i.e., dmPFC, sACC, precuneus) and lateral regions including the dorsolateral PFC and insula, thus pointing to an inability to appropriately deactivate (or disengage) from the resting state in response to stimulus-induced activity (Grimm et al., 2009b). At rest, the reduced negative bold response in MDD patients has also been found to correlate with symptom severity and hopelessness in the vmPFC and dorsal PCC, further showing a disrupted pattern of processing and modulating negative emotions (Grimm et al., 2009a).

Grimm et al. (2008) report hemispheric differences with a hypoactivation in the left dlPFC in response to emotional judgment of negative pictures and increased activity in the right insula and right dlPFC, with the latter two regions being involved in attentional modulation to an emotional judging task, particularly with negative stimuli. This dysregulated pattern of neural activity provides evidence for maladaptive processing and response to emotional events. Indeed, MDD patients exhibit greater difficulty in identifying and evaluating the context of an emotion, whereby they tend to suppress aversive events rather than use more adaptive emotional regulation strategies, such as reappraisal (Abler et al., 2007). These pathological cognitions and attitudes, along with functional changes in limbic and affective processing areas in MDD may account for the behavioral expression of anhedonia, negative mood, and a bleak outlook of future events (Bermpohl et al., 2009).

A recent meta-analysis by Delaveau et al. (2011) reviewing emotional processing in antidepressant-treated MDD patients revealed decreased activation in limbic areas in response to negative stimuli (e.g., right anterior insula, right hippocampus, left amygdala, ventral ACC, and left parahippocampal gyrus). Moreover, the anterior cingulate and bilateral anterior insula were also shown to have greater activation in response to positive and negative stimuli, implying a regulated neural network with the antidepressant treatment (Delaveau et al., 2011). It is noteworthy that the insula was activated to both positive and negative stimuli. The authors posit that these findings may be due to physiological noise (i.e., heartbeat, breathing). However, Fitzgerald et al. (2008) has also found decreased insula activation following antidepressant treatment, lending credence to an improvement in emotional processing and responding in the insula and other limbic regions following antidepressant treatment. These analyses point to an improved response and representation of emotionally laden stimuli in limbic brain areas (Critchley, 2009), leading to a better coordination of interoceptive input and cortical interpretation of emotionally salient information. Given the vastly attributed role of the insular cortex to visceral-sensory information and the conscious emotional representation from these bodily states (Craig, 2002, 2003; Critchley, 2005; Medford and Critchley, 2010), the insula’s functions will be further examined in MDD pathology across various study paradigms.

## Insular Cortex Activity in MDD

### Resting state

Paradigms exploring the brain at rest (e.g., staring at a fixation cross) provide a “state of the affairs” perspective of the neural baseline in MDD. To our knowledge, only two studies have explicitly examined insular activity in depressed populations using a resting state paradigm. The resting state in medication-free MDD patients has been shown to have a decreased functional connectivity in the left insula and amygdala with the resting state network (Veer et al., 2010; see Table 1). Given the insula’s posited role in emotional-related functions, conscious awareness and arousal (Critchley et al., 2004), the altered connectivity between this brain region and the amygdala suggests impaired modulation of negatively valenced or arousing stimuli. This dysregulated cortico-limbic network leaves MDD patients with an inability to properly regulate their mood which may lead them to engage in negative and internally focused cognitions.

**Table 1:** **Imaging studies using a resting state paradigm to examine functional and structural abnormalities in major depressive disorder (MDD)**.

Study authors	Population	Paradigm	Imaging results
**RESTING STATE**			
Hamilton et al. (2011)	MDD patients (*n* = 17, 10 female, 7 male). Mean age (±SD): 45 years ± 3	One fMRI scan was taken at rest (eyes closed)	↓ Activity in the right fronto-insular cortex in MDD patients in the DMN
	Healthy controls (*n* = 17). Mean age (±SD): 42 years ± 2	Observed default mode (DMN) and task-positive network (TPN) activations	↑ Activity in the right fronto-insular cortex in healthy controls in the TPN
Veer et al. (2010)	Recently diagnosed (< 6mo), medication-free MDD patients (*n* = 19, 11 female, 8 male). Mean age (±SD): 36 years ± 10	Resting-state fMRI scan (eyes closed)	↓ Functional connectivity between the left insula and the resting state network in MDD patients
	Healthy controls (*n* = 19, 11 females, 8 males). Mean age (±SD): 36 years ± 11	
**REGIONAL HOMOGENEITY (ReHo)**			
Yao et al. (2009)	MDD in-patients (*n* = 22, 12 female, 10 male). Mean age (±SD): 38 years ± 10	Resting scan (eyes closed)	↓ ReHo in right insula, ACC/PCC in MDD patients
	Healthy controls (*n* = 22, 12 female, 10 male). Mean age (±SD): 39 years ± 11		Anxiety severity, retardation, and hopelessness all positively correlated with ReHo in the right insula
Liu et al. (2010)	First episode un-medicated MDD patients (*n* = 15, 7 female, 8 male). Mean age (±SD): 29 years ± 14 First degree relatives (*n* = 15, 8 female, 7 male). Mean age (±SD): 38 years ± 11	Resting scan (eyes closed)	↓ ReHo in right insula in MDD patients compared to healthy controls ↓ ReHo in right insula in first degree relatives compared to healthy controls		
	Healthy controls (*n* = 15, 7 female, 8 male). Mean age (±SD): 30 years ± 12	
Guo et al. (2011)	Treatment resistant MDD patients (*n* = 24, 12 female, 12 male). Mean age (±SD): 38 years ± 10	Resting scan (eyes closed)	↓ ReHo in left insula in treatment resistant MDD patients compared to healthy controls
	Healthy controls (*n* = 19, 9 female, 10 male). Mean age (±SD): 38 years ± 10	
**STRUCTURAL MRI**			
Lee et al. (2011)	MDD patients (*n* = 47, 42 female, 5 male). Mean age (±SD): 46 years ± 9 Healthy controls (*n* = 51, 45 female, 6 male). Mean age (±SD): 46 years ± 9	Structural MRI scans were obtained to detect structural abnormalities using voxel-based morphometry (VBM)	↓ Decreased gray matter concentration was found the insular gyrus (among other limbic, cerebellar, and frontal regions) in MDD patients compared to healthy controls
			Gray matter concentration in the left insular gyrus was negatively correlated with illness duration in MDD patients compared to healthy controls

*Imaging results only include those relating to insular activity*.

Hamilton et al. (2011) sought to explore DMN dominance in association with maladaptive ruminative behavior in MDD patients. The right fronto-insular cortical (RFIC) network was found to be less activated during the resting state in MDD patients, whereas the opposite was found in healthy controls (i.e., the right fronto-insular cortex was activated in the resting state; see Table 1). The decreased RFIC activation during DMN dominance in MDD patients was also correlated with greater maladaptive rumination. This suggests the possibility that MDD patients may not properly disengage from their internal and/or external environment, and thus, are more likely to negatively process this information in the resting state (Hamilton et al., 2011). These studies point to a disrupted cortico-limbic network in the resting state involving the insula. Hence, is it possible that the faulty integration of information between these regions may increase attention to negative thought patterns, leading to maladaptive rumination.

### Regional homogeneity

Regional homogeneity is a non-parametric analysis measure used to calculate local connectivity within a cluster, which is based on time course BOLD signal correlations between a voxel and its neighbors (Zang et al., 2004). Research has shown reduced ReHo in the insula of first episode un-medicated MDD patients and treatment resistant patients (Liu et al., 2010; Guo et al., 2011; see Table 1). Yao et al. (2009) further report a significant ReHo decrease in the right insula which is further distributed to the orbitofrontal cortex and right ventral and left dorsal ACC, and PCC regions of MDD patients. This reduced connectivity was also positively correlated with anxiety and hopelessness (Yao et al., 2009). As such, the behavioral outcomes may be due to the impairment seen in the cognitive and emotional regulation centers of the brain commonly seen in MDD. These may lead the patients to improperly attend to and modulate incoming information in a pathological way, further exacerbating depressive symptomatology. Interestingly, first degree relatives of MDD patients also show a decreased ReHo in the right insula (Liu et al., 2010), which may indicate increased risk of developing a mood disorder. Given the cognitive and emotional deficits seen in MDD pathology, these data suggest a disrupted pattern of connectivity within the insular cortex extending to frontal and limbic regions.

In fact, voxel-based morphometry, a structural measurement of gray and white brain matter density, reveals that in MDD patients, gray matter concentration is decreased in the limbic system, the insular gyrus, and dorsal raphe nuclei (Lee et al., 2011). The decreased gray matter in these structures has also been negatively correlated with illness duration (Lee et al., 2011). It should be noted that dorsal raphe nuclei neurons project to limbic and cognitive brain regions, using serotonin as its main neurotransmitter (which is hypo-responsive in MDD). The structural abnormalities coupled with impaired functional connectivity in the cortico-limbic system are consistent with the dysregulated neural network seen in MDD pathology. However, successful therapeutic strategies have been shown to protect against gray matter reduction in limbic areas (Phillips et al., 2012).

### Treatment response

In order to evaluate the neural networks involved in depressive pathology, Lui et al. (2011) scanned patients prior to receiving antidepressant treatment, and evaluated them on treatment responsivity after a period of 6 weeks. Non-refractory, meaning patients having shown greater than 50% symptom reduction on the HDRS, had a distributed decreased connectivity among mood regulating brain regions (i.e., amygdala, ACC, bilateral prefrontal cortex, and insula) compared to healthy controls (Lui et al., 2011; see Table 2). Moreover, compared to the refractory group, the non-refractory MDD patients had decreased connectivity in the amygdalar-cingulate and the insular-cingulate regions.

**Table 2:** **Imaging studies using a treatment-based study paradigm in patients with MDD**.

Study authors	Population	Paradigm	Imaging results
**ANTIDEPRESSANT TREATMENT RESPONSIVITY**			
Lui et al. (2011)	Un-medicated MDD patients, HAMD ≥ 18 on day of scan (*n* = 60) Mean age (±SD): Refractory (*n* = 28, 10 female, 18 male): 33 years ± 11 Non-refractory (*n* = 32, 11 female, 21 male): 32 years ± 10 Healthy controls (*n* = 48, 17 female, 31 male). Mean age (±SD): 35 years ± 12	Participants underwent a baseline resting scan (eyes closed) and MDD patients were placed in one of 3 antidepressant medication conditions (tricyclics, SSRIs, SNRIs)	Non-refractory patients had a more distributed ↓ connectivity among ACC, bilateral PFC, HPC, amygdala, and bilateral insula compared to controls ↓Connectivity within the left amygdala/ACC and right insula/precuneus circuits in non-refractory MDD patients compared to controls
**TRANSCRANIAL BRAIN STIMULATION (TMS)**			
Kito et al. (2011)	Treatment resistant MDD patients (*n* = 26, 12 female, 14 male). Mean age (±SD): 46 years ± 14	A PET scan was taken before and after TMS treatment (eyes closed). TMS included stimulation of the right dorsolateral prefrontal cortex (12 sessions over 3 weeks)	↓ rCBF in anterior and posterior insula following low-frequency TMS ↓ rCBF in anterior insula correlated with therapeutic efficacy of low-frequency TMS
Martinot et al. (2011)	Treatment resistant MDD patients (*n* = 31). Mean age (±SD): Responders (*n* = 17, 11 female, 6 male): 49 years ± 6 Non-responders (*n* = 14, 9 female, 5 male): 46 years ± 9 Healthy controls (*n* = 39, 11 female, 8 male). Mean age (±SD): 45 years ± 11	Participants underwent a baseline resting scan (eyes closed) and were placed in one of 3 TMS conditions: PET-guided TMS Active standard Sham-standard TMS	Compared to healthy controls, non-responders to TMS treatment had ↓glucose reuptake in the left ACC, bilateral insula, and frontal regions (dorsolateral prefrontal cortex, orbitofrontal cortex)		

*Imaging results only include those relating to insular activity*.

These findings are surprising given that refractory patients, who showed a poor response to treatment, had increased connectivity in brain regions important for regulating mood states compared to MDD patients having successfully responded to antidepressant treatment. In fact, the refractory patients showed decreased connectivity in mainly prefrontal and thalamic areas in addition to limbic regions (Lui et al., 2011). As such, the authors posit that conventional antidepressants target mostly limbic structures, thus explaining lack of treatment response in the refractory group who showed a greater impairment in the frontal brain regions which regulate and modulate emotional and mood related information from the limbic system.

Transcranial magnetic stimulation therapy is a non-invasive technique used typically in MDD patients otherwise not responding to conventional medication (Kim et al., 2009). TMS operates on the premise that applying a series of pulses to prefrontal-cingulate cortical regions will lead to synaptic plasticity, and strengthen the connections within this circuit, thus alleviating depressive symptoms (Paus and Barrett, 2004). Kito et al. (2011) found that low-frequency stimulation of the right dlPFC lead to decreased regional cerebral blood flow (rCBF) in the right anterior and posterior insula among other prefrontal and cingulate brain regions in the right hemisphere. The decrease in rCBF in the right insula also correlated with therapeutic efficacy of the TMS treatment of treatment resistant MDD patients (Kito et al., 2011; see Table 2). These brain regions have been previously shown to be interconnected, forming a fronto-cingulo-limbic circuit (Seminowicz et al., 2004), and other brain stimulation studies have also shown that stimulating parts of this circuit (i.e., subgenual ACC) help in alleviating the clinical symptoms of depression (Mayberg et al., 2005; Puigdemont et al., 2012).

In line with the TMS findings described above, using FDG-PET, Martinot et al. (2011) also found decreased glucose uptake in prefrontal cortical regions and in the anterior cingulate cortex in both responders and non-responders to high-frequency repetitive TMS treatment in the prefrontal cortex. Particularly, non-responders had lower levels of glucose reuptake in the left ACC, bilateral insula, and left orbitofrontal and dorsolateral PFC (Martinot et al., 2011). In addition, non-responders also had lower gray matter volume in the left ACC, and prefrontal cortical regions (orbitofrontal, ventro-, and dorsolateral PFC; Martinot et al., 2011). While lower rCBF in the insula has been reported to correlate with successful TMS outcomes (Kito et al., 2011), it seems that a reduced volume in cognitive and emotional regulation brain areas coupled with hypometabolism in these regions, fails to exert the beneficial effects otherwise seen with TMS.

### Emotional processing

One of the main clinical symptoms in MDD pathology is the bias toward stimuli of negative valence. The inability to suppress the increased attention to these stimuli causes a disruption in normal emotional processing. In fact, Herwig et al. (2010) allude to a “pessimistic” tendency whereby MDD patients show similar neural activity when anticipating an emotional picture of unknown valence as they do when expecting negatively valenced pictures. Studies have also demonstrated increased neural activity in the insula of MDD patients viewing expressions of disgust (Sprengelmeyer et al., 2011; see Table 3), even when these were morphed to 50% of their original intensity (Surguladze et al., 2010). This may be due to decreased gray matter concentrations in the insula of MDD patients (Sprengelmeyer et al., 2011). Indeed, symptom severity has been negatively correlated with gray matter volume in bilateral insula (Sprengelmeyer et al., 2011).

**Table 3:** **Imaging studies using emotionally based paradigms to examine functional and structural abnormalities in major depressive disorder (MDD)**.

Study authors	Population	Paradigm	Imaging results
**EMOTIONAL-BASED TASKS**			
Herwig et al. (2010)	Medicated MDD in-patients (*n* = 14, 8 female, 6 male). Mean age (±SD): 40 years ± 11	fMRI cueing task: Known condition (cue was presented showing emotional valence of picture)	↑ bilateral insula activation in response to pending picture presentation with unknown emotional valence
	Healthy controls (*n* = 14, 8 female, 6 male). Mean age (±SD): 28 years ± 4	Unknown condition (absence of cues)	Positive correlation between depressive symptoms and right insula during unknown and negative picture expectation
Surguladze et al. (2010)	MDD patients (*n* = 9, 4 female, 58 male). Mean age (±SD): 43 years ± 7	Two 6-min experiments: photos showing expressions of disgust and neutral faces	↑ activation in the left insula in response to expressions showing 50 and 100% disgust, compared to neutral expressions
	Healthy controls (*n* = 9, 4 females, 5 males). Mean age (±SD): 40 years ± 15	Fearful expressions and neutral faces Participants were tested on accuracy of labeling the facial expressions.	
Samson et al. (2011)	Un-medicated MDD patients (*n* = 21, 7 female, 14 male). Mean age (±SD): 41 years ± 9 Healthy controls (*n* = 12, 4 female, 8 male). Mean age (±SD): 36 years ± 11	Emotional paradigm: passively view sad facial expressions and neutral pictures (homes in Munich) fMRI: MDD patients were imaged in the drug free state and 4 weeks following the start of drug administration	Negative correlation between depressive symptom severity and bilateral insula activation of MDD patients following drug treatment when viewing sad facial expressions compared to the neutral pictures
Demenescu et al. (2011)	MDD out-patients (*n* = 59, 39 female, 20 male). Mean age (±SD): 36 years ± 11	Presented color facial expressions (angry, fearful, sad, happy neutral, scrambled faces)	↑ activation in the right insula in response to happy vs. scrambled faces in MDD patients compared to healthy controls
	Anxiety disorder out-patients (*n* = 57, 43 female, 24 male). Mean age (±SD): 36 years ± 9	
	MDD/Anxiety out-patients (*n* = 66, 43 female, 23 male). Mean age (±SD): 36 years ± 11	Participants were asked to rate the gender of each picture	
	Healthy controls (*n* = 56, 34 female, 22 male). Mean age (±SD): 40 years ± 10	
Sprengelmeyer et al. (2011)	Medicated MDD patients (*n* = 15, 6 female, 9 male). Mean age (±SD): 46 years ± 11	Experiment 1: facial recognition of emotion + morphed faces on a continuum. Participants had to attribute the emotion to the face	Experiment 1: average discrimination accuracy for MDD patients was positively correlated with gray matter volume in bilateral anterior insula
	Healthy controls (*n* = 15, 6 female, 9 male). Mean age (±SD): 46 years ± 11	Experiment 2: facial recognition at 4 different intensities (20, 40 60, 80%). Participants had to press a key as soon as they identified the emotion	Experiment 2: ↓ gray matter volume in insular cortex in MDD patients
Townsend et al. (2010)	Un-medicated MDD patients (*n* = 15, 6 female, 9 male). Mean age (±SD): 46 years ± 11 Healthy controls (*n* = 15, 6 female, 9 male). Mean age (±SD): 45 years ± 12	Face matching paradigm: 3 conditions: Match emotions Identify emotion Match forms	No significant insular activation in MDD patients ↑ right insula activation in healthy controls in face matching compared to form matching

*Imaging results only include those relating to insular activity*.

Increased insular activation in MDD patients is not limited to negative stimuli. It has also been detected when processing happy facial expressions compared to scrambled faces (Demenescu et al., 2011; see Table 3). Curiously, no significant neural activations were found with fearful or angry facial expressions (Demenescu et al., 2011). However, it should be noted that this sample consisted of MDD out-patients with mild to moderate depressive symptomatology, suggesting a relatively well-functioning population. Townsend et al. (2010) also failed to find neural activation in the insula in a face matching paradigm in MDD patients, although they found increased insular activity in healthy controls. This may have resulted from using a non-medicated MDD sample population, whereby altered cortico-limbic connectivity can pose a certain degree of difficulty in processing and interpreting emotionally based stimuli. Indeed, Samson et al. (2011) have demonstrated improved regulation and modulation of emotional content in the insular region following a 4-week antidepressant treatment. As such, impairments in limbic regions responsible for the automatic processing of emotionally salient information may lead to aberrant insular activity simply by arousing the system. This will impact insular function (i.e., interoceptive state) given the strong interconnectivity between the amygdala and insular cortex (Shelley and Trimble, 2004).

### Cognitive, memory, and sensory paradigms

Given the clinical symptoms associated with depressive pathology (e.g., anhedonia, impaired concentration, and working memory deficits), task-related paradigms aim to provide insight into the neural alterations associated with symptomatology in MDD patients. Videbech et al. (2003) explored cognitive impairments using a phonological verbal fluency task in an adult sample of MDD patients. Participants underwent a PET scan at rest and while performing the task (i.e., “name as many words with the letter ‘T”’). Compared to the resting state, MDD patients showed greater activation in the left insula while performing the task, as well as in anterior cingulate and frontal cortex (Videbech et al., 2003; see Table 4). The greater metabolic activity in these regions insinuates that MDD patients have greater difficulty in recruiting the mental resources to perform this task. Indeed, one must use semantic memory in recalling words starting with a specific letter, and a certain level of organization is required to quickly perform this task while generating a large list of words. The decreased activation in the insula, which integrates information from multiple sensory modalities and converges onto executive brain regions, may explain the recall-related impairments and overall difficulty in engaging in mental processing in MDD patients (Nagai et al., 2007).

**Table 4:** **Imaging studies using task-related paradigms to examine functional and structural abnormalities in major depressive disorder (MDD)**.

Study authors	Population	Paradigm	Imaging results
**COGNITIVE, MEMORY, AND SENSORY**			
Videbech et al. (2003)	MDD in-patients with a HDRS score ≥ 17 (*n* = 41, 29 female, 12 male). Mean age (±SD): 42 years ± 13	PET scan taken at rest	↓ Activity in the left insula in MDD patients when comparing verbal fluency to the resting state
	Healthy controls (*n* = 46, 30 female, 16 male). Mean age (±SD): 41 years ± 12	PET scan taken during phonological verbal fluency task. fMRI scan used as a template for spatial normalization of PET data	↑ Activity in the bilateral insula in healthy controls when comparing verbal fluency to the resting state
Young et al. (2011)	Un-medicated MDD patients in a current depressive episode (*n* = 12, 8 female, 4 male). Mean age (±SD): 34 years ± 11	Autobiographical memory task (fMRI): Cue words were presented (positive, negative, neutral)	↓ BOLD signal in the anterior insula during memory recall in both groups, however, greater reduction in MDD patients
	Healthy controls (*n* = 14, 7 females, 7 males). Mean age (±SD): 29 years ± 9	Participants pressed a button once a memory was recalled and rated it on valence, arousal, and recency	↓ BOLD signal in bilateral anterior insula and posterior insula during autobiographical memory recall in both groups (magnitude of reduction greater in MDD patients)
Strigo et al. (2010)	Un-medicated MDD patients for a minimum of 30 days (*n* = 15, 12 female, 3 male). Mean age (±SD): 25 years ± 6 Healthy controls (*n* = 17, 10 females, 7 males). Mean age (±SD): 24 years ± 5	fMRI: 4 conditions: Painful heat − task Painful heat + task Non-painful warmth − task Non-painful warmth + task	When shifting stimulus intensity (1 vs. 3 and 2 vs. 4), MDD patients show ↓ activation in the right anterior insula and bilateral mid-insula When shifting cognitive demand (1 vs. 2 and 3 vs. 4) and both stimulus intensity and cognitive demand (2 vs. 3 and 1 vs. 4), MDD patients show ↓ activation in the right mid-insula

*Imaging results only include those relating to insular activity*.

Using an autobiographical memory paradigm, Young et al. (2011) examined un-medicated MDD patients’ ability to recall personal events associated with positive, negative, and neutral cue words. Lower BOLD signals were detected in the dorsolateral PFC, cingulate, and limbic regions, including the posterior and bilateral anterior insula during memory recall irrespective of word valence in depressed patients (Young et al., 2011; see Table 4). These same insular regions showed lower activation in response to specific autobiographical memory recall, whereby MDD patients recalled fewer positive memories compared to healthy controls (Young et al., 2011). These results extend Videbech’s et al. (2003) findings of lower insular activation, albeit in response to recalling general memories and those associated with personally relevant experiences. Given that depressed patients retrieved less specific autobiographical memories, which were positive in nature, suggests a diminished sensitivity and attention to this type of stimuli and is further supported by decreased activity in the insula.

Major depressive disorder patients also show impairments in adapting to different environmental or homeostatic states. Strigo et al. (2010) evaluated shifts in anticipatory responses to stimuli that were either cognitive (task vs. no task), sensory (moderately painful heat vs. non-painful warmth), or a combination of both. When anticipating a shift in stimulus intensity (i.e., heat vs. warmth), un-medicated MDD patients showed lower BOLD signal activity in the right anterior insula and bilateral mid-insula, among prefrontal, parietal, temporal, and cerebellar regions (Strigo et al., 2010; see Table 4). On the other hand, shifts in stimuli that were cognitive in nature or a combination of both cognitive and sensory elicited a decreased activation in the right mid-insula and medial temporal gyrus. In MDD patients, an inability to generate positive feelings or excitement when thinking about future events substantiates the negative bias common in mood disorders, whereby increased focus is placed on negative stimuli. This lack of emotional reactivity when anticipating events of different sensory salience indicates integration impairments between environmental stimuli and the sensory/affective processing of these events. As well, the decreased insular activity may imply that the response to the external environmental cues may not be properly modulated, leading to a faulty integration of this information with interoceptive states (Critchley, 2009).

## Conclusion

Major depressive disorder is associated with deficits in emotional processing, with a bias toward negative stimuli. This may be due to the fronto-limbic alterations in MDD pathology, whereby limbic regions are hypoactivated and frontal executive regions show decreases in activity. As such, the top-down modulation of emotionally based information is impaired, leading to depressive symptomatology (e.g., rumination). These deficits in emotional processing could be further exacerbated by the morphological changes (e.g., gray matter volume reductions in fronto-limbic structures) and neurochemical imbalances in the brain.

Recently, much attention has focused on the neural basis of depressive pathology, particularly with respect to the baseline resting state in MDD patients. Disrupted connectivity in fronto-limbic structures is commonly reported as are abnormal deactivations in default mode regions. This supports the on-going mental processing of emotionally based information in MDD patients who have great difficulty disengaging from internal and external input. Of interest, the insular cortex with its many functions in emotional, sensorimotor and interoceptive processing, and extensive connectivity with DMN regions, also has been implicated in MDD pathology.

Review of recent literature points to decreases in connectivity of the insula with the amygdala and default mode regions (anterior and posterior cingulate cortex). As well, the insula shows greater activity in the resting state, supporting the inability of MDD patients to disengage from externally cued events, which may lead to pathological self-focused mental ruminative behaviors. However, therapeutic brain stimulation studies have shown positive results in regulating insular activity by decreasing its activation in MDD patients, pointing to an effective therapeutic strategy in re-establishing functional emotional processing.

Differing results have been obtained when looking at the processing of cognitive and emotionally based information in the insular region of MDD patients. Specifically, cognitive-based tasks requiring working memory, retrieval, and active processing showed decreases in the insula which may suggest impairments in integrating various cognitive processes required for mentally demanding activities. On the other hand, studies using emotionally based paradigms showed increases in insular activity. These findings may be due to the MDD patients’ greater susceptibility in attending to emotionally arousing stimuli. Indeed, greater insular activity was found primarily in response to negative emotions, pointing to impaired processing and regulation of negative mood and emotions.

## Conflict of Interest Statement

The authors declare that the research was conducted in the absence of any commercial or financial relationships that could be construed as a potential conflict of interest.
